# Effects of serum from breast cancer surgery patients receiving perioperative dexmedetomidine on breast cancer cell malignancy: A prospective randomized controlled trial

**DOI:** 10.1002/cam4.2654

**Published:** 2019-10-30

**Authors:** Yan Liu, Jiaxin Sun, Tong Wu, Xiaoying Lu, Yueyao Du, Hongwei Duan, Weifeng Yu, Diansan Su, Jinsong Lu, Jie Tian

**Affiliations:** ^1^ Department of Anesthesiology Renji Hospital Shanghai Jiao Tong University School of Medicine Shanghai China; ^2^ Department of Anesthesiology Shanghai Pudong Hospital Fudan University Pudong Medical Center Shanghai China; ^3^ Department of Breast Renji Hospital Shanghai Jiao Tong University School of Medicine Shanghai China

**Keywords:** breast cancer, cell malignancy, dexmedetomidine, general anesthesia

## Abstract

Adrenergic receptors (ARs) have gained attention for their involvement in breast cancer (BC) progression. Dexmedetomidine, a selective α_2_‐AR agonist, has been reported to increase the malignancy of BC cells in vitro or stimulate tumor growth in mice. However, clinical evidence is lacking. Clinical research in this area is important as dexmedetomidine is widely used in BC surgery patients. Here we allocated 24 women with primary BC to the dexmedetomidine group (who received a total dose of 2 μg kg^−1^ dexmedetomidine perioperatively) or to the control group (who received the same volume of normal saline). Venous blood was obtained from all patients immediately upon entering the operating room and 24 hours postoperatively. Serum was then exposed to MCF‐7 cells at a concentration of 10% for 24 hours. Cell proliferation, migration, and invasion were analyzed using EdU, Transwell, and Matrigel methods, respectively. We found that postoperative serum from those who received dexmedetomidine was associated with significantly increased cell proliferation, migration, and invasion compared with preoperative serum when used to culture MCF‐7 cells. The mean percentage change from post to preoperative values in these cell functions was significantly larger in the dexmedetomidine group than in the control group (proliferation, 30.44% vs 8.45%, *P* = .0024; migration, 15.90% vs 3.25%, *P* = .0015; invasion, 8.17% vs 2.13%, *P* = .04). In conclusion, these findings suggest that in patients undergoing surgery for primary BC, perioperative administration of dexmedetomidine might influence the serum milieu in a way that favors the malignancy of MCF‐7 cells.

Clinical trial registration: NCT03108937.

## Graphical abstract

Exposure of MCF‐7 cells to postoperative serum from patients receiving dexmedetomidine during surgery, but not control saline, had significantly higher proliferation, invasion, and migration than preoperative serum. The mean percentage changes from post to preoperative values in all these cellular malignant properties were also significantly larger in the Dex group compared with the control group. These findings suggest that the utilization of Dex during breast cancer surgery may modulate the patients' serum milieu to a direction that favors the malignancy of MCF‐7 cells.

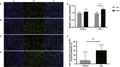

## 1 INTRODUCTION

Breast cancer (BC) is one of the most common tumors among females, which affects about 12% of women worldwide.^1^ In 2018, BC alone is projected to account for 30% of all new cancer diagnosed in women in the United States.^2^ Meanwhile, BC was also the most frequent cause of death in 11 regions of the world; approximately 15% of deaths among women worldwide were attributed to BC.^3^ Postoperative local recurrence and distant metastasis are the foremost concerns of patients and their caregivers. Many advances have been made in understanding the mechanisms involved in the recurrence and metastasis of BC.

The activation of adrenergic receptor (AR) has attracted great attention as a regulator of cancer progression in recent years. ARs are classically classified into two main groups, α‐AR and β‐AR, both of which are widely distributed in most of mammalian tissues.^4^ Powe and colleagues performed immunohistochemistry on tissue microarrays to characterize AR expression in operable breast tumors and demonstrated that expression of α‐AR and β‐AR is associated with poor clinical outcome in BC.^5^ Particularly, numerous evidence confirm that α_2_‐AR plays an essential role in BC progression. The protein is overexpressed in tumors with a more malignant phenotype characteristic and its expression correlates with the risk of BC relapse.^6^ In mice, pharmacologic activation of α_2_‐AR enhances mammary tumor growth.^7^ Consistently, in in vitro studies, cell proliferation and migration capacity are increased when mammary tumor cells are treated with specific α_2_‐AR agonists, such as clonidine.^4^, ^8^ Furthermore, the α_2_‐AR antagonist rauwolscine reverses the effects of the agonists and inhibits cell proliferation and tumor growth.^7^, ^8^, ^9^

Dexmedetomidine (Dex) is a highly selective α_2_‐AR agonist with sedative, analgesic, and anesthetic properties. It is widely used in the ICU and anesthesia department due to its unique ability of inducing a calm state with no respiration depression. However, does Dex affect prognosis in BC patients after activating the α_2_‐AR? Several laboratory studies have shown that stimulation by Dex increases BC cell proliferation and migration and tumor growth.^10^, ^11^, ^12^ However, clinical evidence is lacking.

The serum milieu, which is a mixture of various molecular products, including chemoattractants, nutrients, growth factors, cytokines, and transcription factors, secreted by the blood cells or the primary tumor, is essential in determining whether recurrence and metastases can be established or grown, because a favorable environment enables tumor cells to transmigrate into vessels and survive in the circulatory system.^13^ To clarify the potential risk of Dex in clinical BC patients, we randomized primary BC surgical patients to receive either Dex or saline during the perioperative period and collected the patients' serum both pre and postoperatively. By comparing the effects of postoperative serum from these patients with BC cell function in vitro, we utilized serum as a marker of the overall effects of Dex on patients' systemic environment. Our results demonstrate that in patients undergoing BC surgery, perioperative utilization of Dex may influence the serum milieu in a way that favors the malignancy of MCF‐7 cells.

## 2 MATERIALS AND METHODS

### 2.1 Study design and patients

This prospective, randomized, and controlled clinical trial was designed in accordance with the CONSORT recommendation and was conducted in Renji Hospital, Shanghai Jiao Tong University School of Medicine, Shanghai, China, between April 2017 and July 2017. It was approved by the Institutional Human Ethics Committee ([2016]037(2), Shanghai, China) of Renji Hospital and is registered at http://www.ClinicalTrials.gov (NCT03108937). Informed consent was obtained from all patients or legally authorized representatives.

Female patients undergoing simple mastectomy combined with sentinel lymph node biopsy or modified radical mastectomy (Auchincloss method) for biopsy‐proven primary BC were enrolled in the study. Inclusion criteria were as follows: (a) female patients; (b) ASA Classes I‐III; (c) aged 18 to 75 years old; and (d) primary tumors (T) 2‐3, regional lymph nodes (N) 0‐2, distant metastasis (M) 0 (ie, tumor > 20 mm in the greatest dimension without known extension beyond the breast and axillary nodes). Patients were excluded if they (a) had previous breast surgery; (b) were diagnosed with inflammatory BC; (c) were addicted to opioids; (d) had serious major mental or physical illness (heart, pulmonary, hepatic, or renal diseases); or (e) were diagnosed with metastatic BC.

### 2.2 Randomization and blindness

Eligible patients were stratified by menopause status and allocated randomly 1:1 to receive Dex or saline as a treatment according to the computer generated codes. The PROC program in SAS (version 9.0, SAS Institute Inc) was used to generate the sample randomization sequence with a 1:1 allocation.

Patients and all study personnel, except the medicine provider, were blinded to the treatment assignment.

### 2.3 Procedures

Patients in both groups received general anesthesia (GA) for the BC surgery. GA was induced with 0.05 mg kg^−1^ of midazolam, 3‐6 μg kg^−1^ of fentanyl, 1‐2 mg kg^−1^ of propofol, and 0.2 mg kg^−1^ of cisatracurium for all patients. Anesthesia was maintained with 0.1‐0.2 μg kg^−1^ min^−1^ of remifentanil, 4‐8 mg kg^−1^ h^−1^ of propofol, and 0.1 mg kg^−1^ h^−1^ of cisatracurium to maintain the BIS value within the range of 40‐60. Lungs were mechanically ventilated to maintain ETCO_2_ at 35‐45 mmHg.

Hypotension (a ≥20% decrease in systolic blood pressure (SBP) from the baseline) was treated using ephedrine. Bradycardia (a heart rate (HR) slower than 50 beats per minute) was corrected with atropine. Hypertension (a ≥20% increase in SBP from the baseline) or tachycardia (an HR faster than 100 beats per minute) was managed by titrating the infusion rate of propofol or remifentanil to achieve an adequate anesthesia depth.

All patients were given 1 μg kg^−1^ of fentanyl before the end of surgery for the management of postoperative pain. Rescue analgesia if needed was triggered by a visual analog scale pain score ≥4, with intravenous injection of 40‐80 mg of parecoxib sodium every 24 hours.

Venous blood was obtained from all patients immediately upon entering the operating room and 24 hours postoperatively. Samples were centrifuged at 3000 rpm for 10 minutes at 4°C and the resulting serum was stored at −80°C for future use.

### 2.4 Intervention

Patients in the Dex group received a loading dose of 1 μg kg^−1^ of Dex 15 minutes before GA induction and then an infusion at a rate of 0.5 μg kg^−1^ h^−1^ for 2 hours during surgery, such that each patient received 2 μg kg^−1^ of Dex in total. Patients in the control group were given the same amount of normal saline.

### 2.5 Data collection

General characteristics were collected in the preoperative interview, reported by the patients themselves. ASA classification and surgical information, including the duration and type of surgery and anesthetic dosages, were obtained through anesthetic records. Cancer characteristics were recorded in accordance with the pathology report.

#### 2.5.1 Primary outcome measures

The primary outcome was the mean percentage change from post to preoperative values of the proliferation of MCF‐7 cells cultured in the patients' serum.

The human BC cell line MCF‐7 was purchased from the Chinese Academy of Sciences. Cells were cultured in phenol red‐free DMEM supplemented with 10% fetal bovine serum (FBS), 100 IU/mL penicillin, and 100 μg/mL streptomycin at 37°C in a humidified 5% CO_2_ atmosphere. EdU incorporation assay was utilized to detect DNA synthesis, which reflects cell proliferation. MCF‐7 cells seeded in 96‐well plates (5 × 10^3^ cells/well) were serum‐starved overnight before incubation in medium containing 10% of patient serum for 24 hours. About 50 μmol/L of 5‐ethynyl‐2ʹ‐deoxyuridine (EdU) was added into the medium and incubated for 2 hours before the end of the cell treatment. The incorporation of EdU into actively proliferating MCF‐7 cells was determined using a Cell‐Light™ EdU DNA Cell Proliferation Detection Kit (RiboBio, China) following the manufacturer's instructions. Nuclei were stained with Hoechst.

All the experiments were performed in triplicate. Digital images were acquired under fluorescence microscopy (Leica, Germany), with an original magnification of 100×, and analyzed using ImageJ software to calculate the percentage of cells positive for EdU (representing S‐phase cells). %EdU + cells = the number of positive EdU cells/ the total number of nuclei × 100%. The values were then normalized to the %EdU + rate obtained with preoperative serum of control patients and presented as a ratio of preoperative control rate. The mean percentage change from post to preoperative values for each individual patient was also calculated and compared between the Dex and control groups. The mean percentage change = [(%EdU + cells with postoperative serum) − (%EdU + cells with preoperative serum)]/(%EdU + cells with preoperative serum) × 100%.

#### 2.5.2 Secondary outcome measures

The secondary outcomes included the mean percentage change from post to preoperative values of migration or invasion of MCF‐7 cells cultured in patients' serum.

The in vitro migration activity of MCF‐7 cells was analyzed using the modified Boyden chamber (6.5 mm in diameter, 8.0 μm pores; Transwell). Cells that are serum‐starved overnight were placed in the upper part of the chamber at a density of 1 × 10^6^ cells/mL in 100 μL serum‐free medium, whereas 600 μL of patients' serum sample at a final concentration of 10% was added to the bottom chamber. After 24 hours coincubation at 37°C, the cells migrating to the lower surface of the membrane were stained with crystal violet, photographed, and analyzed using a fluorescence microscope.

The invasive potential of the cells was analyzed using a Matrigel‐coated modified Boyden chamber. Polycarbonate membranes were spread with 50 μL of Matrigel (BD Biosciences) on the upper surface of Transwell cell culture chambers and incubated at 37°C for 30 minutes. Then, cells were incubated in the upper chamber and patients' serum diluted with medium to a final concentration of 10% was added to the lower chamber. After incubation at 37°C for 24 hours, the number of cells that invaded the lower side of the upper chamber was counted.

For both migration and invasion assays, eight fields per chamber were counted and the average was calculated to reflect the migration or invasion activity of the sample. The values were then normalized to the calculated average cell number obtained with preoperative serum of control patients and presented as a ratio of the preoperative control number. The mean percentage change from post to preoperative values for each individual patient was also calculated and compared between the Dex and control groups. Mean percentage change = [(No. of migrated/invaded cells with postoperative serum) − (No. of migrated/invaded cells with preoperative serum)]/(No. of migrated/invaded cells with preoperative serum) × 100%.

### 2.6 Data analysis

Pass (version 11.0, NCSS, LLC) software was used for the sample size calculation. The test for two means (two‐sample *t* test) was used. Based on previous studies,^10^, ^11^, ^12^ assuming the Dex treatment will result in a 15% increase in the mean percentage change from post to preoperative proliferation, with an SD of 10%, the study will require 11 patients in each group to have a 90% power with an α equal to 0.05. If the attrition rate was set at 15%, 13 patients per group should be included.

Data were expressed as mean ± SD. The SPSS 24.0 software package (SPSS Inc, Chicago, USA) was adopted for analysis and GraphPad Prism 5 (GraphPad Software, Inc, La Jolla, CA) was used for plotting graphical representations of data. When comparing the cell proliferation, migration, and invasion stimulated by pre and postoperative serum from the two groups, multiple comparisons were performed using two‐way ANOVA followed by Dunnett's T3 post hoc test. The mean percentage change between the two groups was analyzed using an independent samples’ *t* test. Enumeration variables, such as ASA physical status, number of patients receiving neoadjuvant chemotherapy (NAC), and the number of estrogen receptor‐positive tumors, were analyzed using Fisher's exact test. Statistical tests were two‐sided and *P* < .05 was considered statistically significant.

## 3 RESULTS

### 3.1 Patients

**Figure 1:**
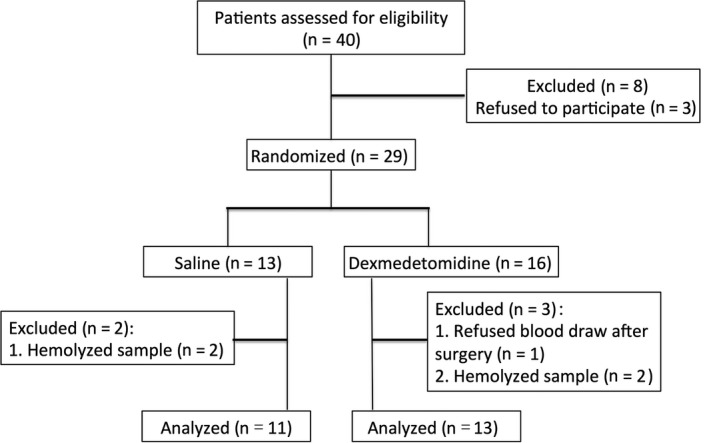
Trial flow diagram. A total of 29 patients were recruited and randomized to the control group or Dex group. Twenty‐four patients were finally included in the analysis, 11 in the control group, and 13 in the Dex group

From April 2017 to July 2017, a total of 29 patients were recruited and randomized to the control or Dex groups. Among them, one refused the blood draw at 24 hours after surgery and four samples had hemolysis. Therefore, 24 patients were finally included in the analysis, 11 in the control group and 13 in the Dex group (Figure 1). The two groups were comparable in the general patient information (Table 1), surgical information, and cancer characteristics (Table 2). The Dex group required fewer total doses of midazolam, propofol, and remifentanil and higher doses of ephedrine and atropine compared with the control group during the surgical procedures, but none of the differences reached statistical significance (Table 2). None of the patients required parecoxib sodium within 24 hours postoperatively.

**Table 1:** General characteristics of 24 mastectomy patients, reported as mean (SD) or number (proportion)

	Control group (n = 11)	Dex group (n = 13)
Age (y)	53.0 (9.31)	56.7 (12.25)
Height (cm)	158.9 (7.20)	159.1 (4.11)
Weight (kg)	62.6 (11.80)	56.8 (6.74)
Menopause, n	6 (55%)	8 (62%)
ASA I/II/III, n	3/8/0 (27/73/0%)	3/10/0 (23/77/0%)
Neoadjuvant chemotherapy, n	3 (27%)	5 (38%)

Abbreviation: ASA, American Society of Anesthesiologists.

**Table 2:** Surgical information and cancer characteristics of patients, reported as mean (SD) or number(proportion)

	Control group (n = 11)	Dex group (n = 13)	*P* value
Duration of surgery(min)	160.8 (39.86)	157.1 (32.93)	0.80
Operation			
Modified radical mastectomy, n	8(73%)	12(92%)	0.30
Simple mastectomy and sentinel lymph node biopsy, n	3(27%)	1(8%)	0.30
Maximum diameter (cm)	2.4 (0.57)	2.6 (0.54)	0.23
ER positive, n	6(55%)	10(77%)	0.39
PR positive, n	9(82%)	10(77%)	1.0
HER2 positive, n	6(55%)	7(54%)	1.0
Tumor stage IIa/IIb/IIIa, n	7/2/2(64/18/18%)	8/4/1(62/31/7%)	0.73
Fentanyl (mg)	0.3 (0.03)	0.3 (0.02)	0.47
Midazolam (mg)	3.1 (0.58)	2.8 (0.34)	0.10
Propofol (mg)	1352.6 (446.97)	1083.8 (232.25)	0.07
Remifentanil (mg)	1.5 (0.40)	1.4 (0.31)	0.95
Ephedrine (mg)	1.1 (2.43)	2.3 (3.04)	0.30
Atropine (mg)	0 (0)	0.06 (0.16)	0.20
Parecoxib sodium (mg)	0	0	1.00

Abbreviations: ER, estrogen receptor; HER2, human epidermal growth factor receptor‐2; PR, progesterone receptor;

### 3.2 Serum from patients receiving Dex facilitated cell proliferation of MCF‐7 cells

**Figure 2:**
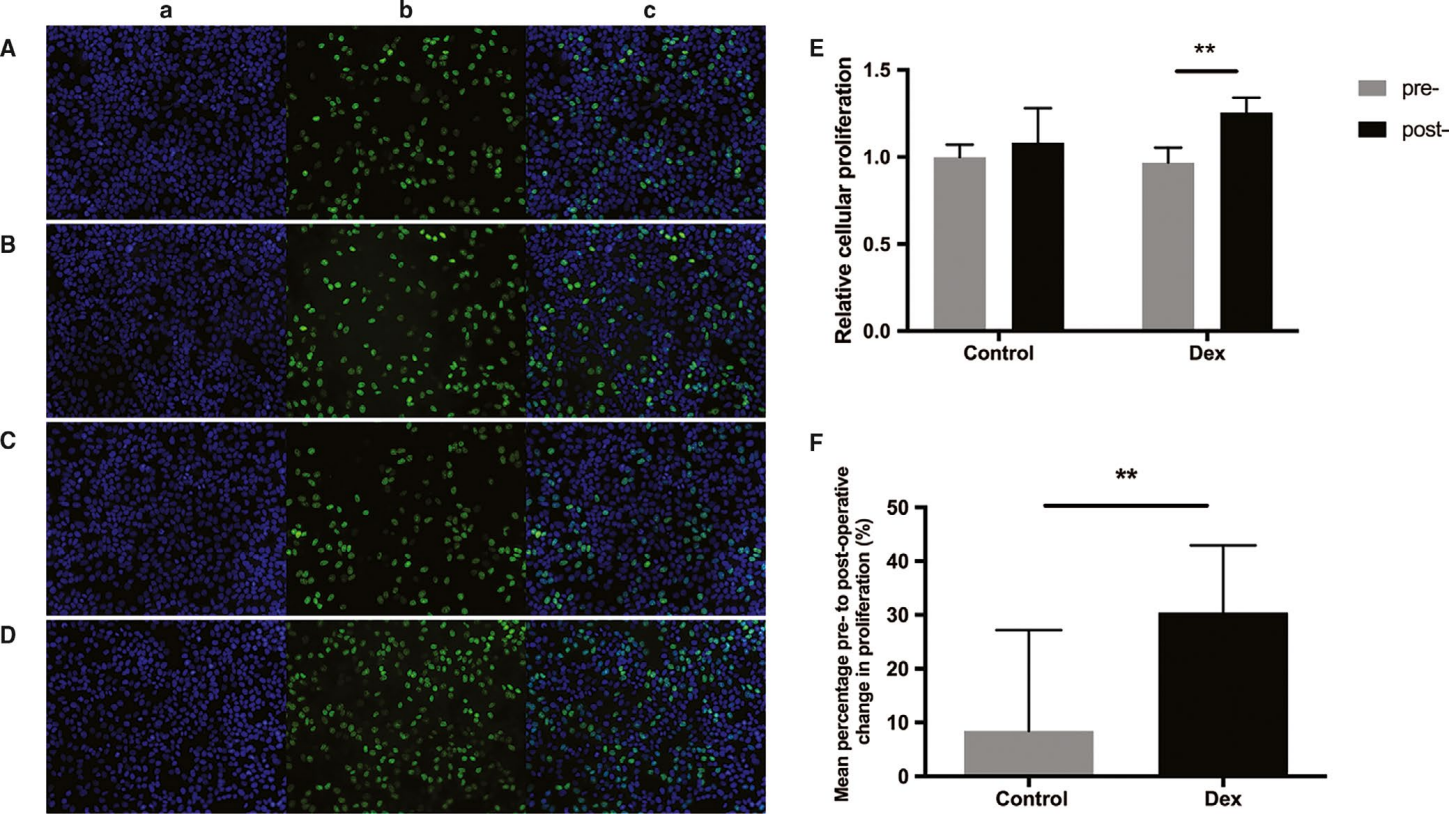
Serum from patients receiving dexmedetomidine increased the proliferation of human MCF‐7 breast cancer cell line. A, Serum from the control group, preoperatively; B, serum from the control group, 24 h postoperatively; C, serum from the Dex group, preoperatively; D, serum from the Dex group, 24 h postoperatively. a, Nuclei were counterstained with Hoechst 33 342 (blue); b, proliferative cells were stained with EdU (green); c, Merge. Original magnification, 100×. E, Graphical representation of EdU positive cell proportion of the four groups. The value obtained with preoperative serum of control patients was considered 1.0, and the ratios relative to this are shown. F, Graphical representation of the mean percentage change from post to preoperative values in EdU positive cells in the Dex group vs the control group. Values were expressed as mean ± SD. n = 11 in the control group. n = 13 in the Dex group. **, *P* < .01. Dex = dexmedetomidine

EdU incorporation assay was utilized to investigate the effects of serum from both groups on the proliferation of MCF‐7 cells. There was no significant difference in the percentage of EdU positive cells between the two groups when incubated with preoperative serum samples (Figure 2A,C,E). In the control group, although the %EdU+ rate increased when cells were treated with postoperative serum compared with preoperative serum, the difference did not reach statistical significance (1.00 ± 0.07 precontrol vs 1.08 ± 0.20 postcontrol, *P* = .08). Conversely, exposure to postoperative serum from patients in the Dex group resulted in a significant increase in proliferation than the corresponding preoperative serum (0.97 ± 0.09 pre‐Dex vs 1.25 ± 0.09 post‐Dex, *P* < .001) (Figure 2E). Furthermore, the mean percentage change from post to preoperative values in cell proliferation was significantly larger in the Dex group compared with the control group (30.44% ± 12.5% in the Dex group vs 8.45% ± 18.73% in the control group, *P* = .0024) (Figure 2F).

### 3.3 Serum from patients receiving Dex facilitated cell migration and invasion of MCF‐7 cells

**Figure 3:**
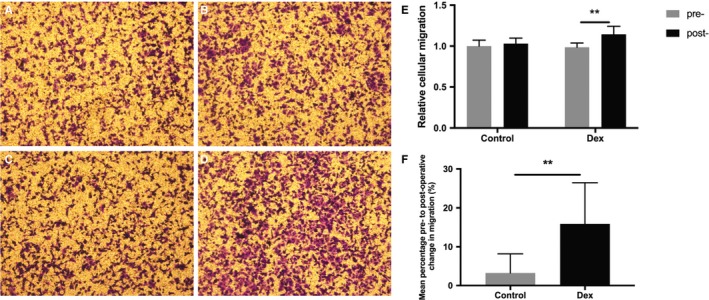
Serum from the dexmedetomidine group promoted the migration of human MCF‐7 breast cancer cell line. A, Serum from the control group, preoperatively; B, serum from the control group, 24 h postoperatively; C, serum from the Dex group, preoperatively; D, serum from the Dex group, 24 h postoperatively. Original magnification, 100×. E, Graphical representation of cells migrated to the lower surface of the four groups. The value obtained with preoperative serum of control patients was considered 1.0 and the ratios relative to this are shown. F, Graphical representation of the mean percentage change from post to preoperative values in migrated cell numbers in the Dex group vs the control group. Values were expressed as mean ± SD. n = 11 in the control group. n = 13 in the Dex group. **, *P* < .01. Dex = dexmedetomidine

**Figure 4:**
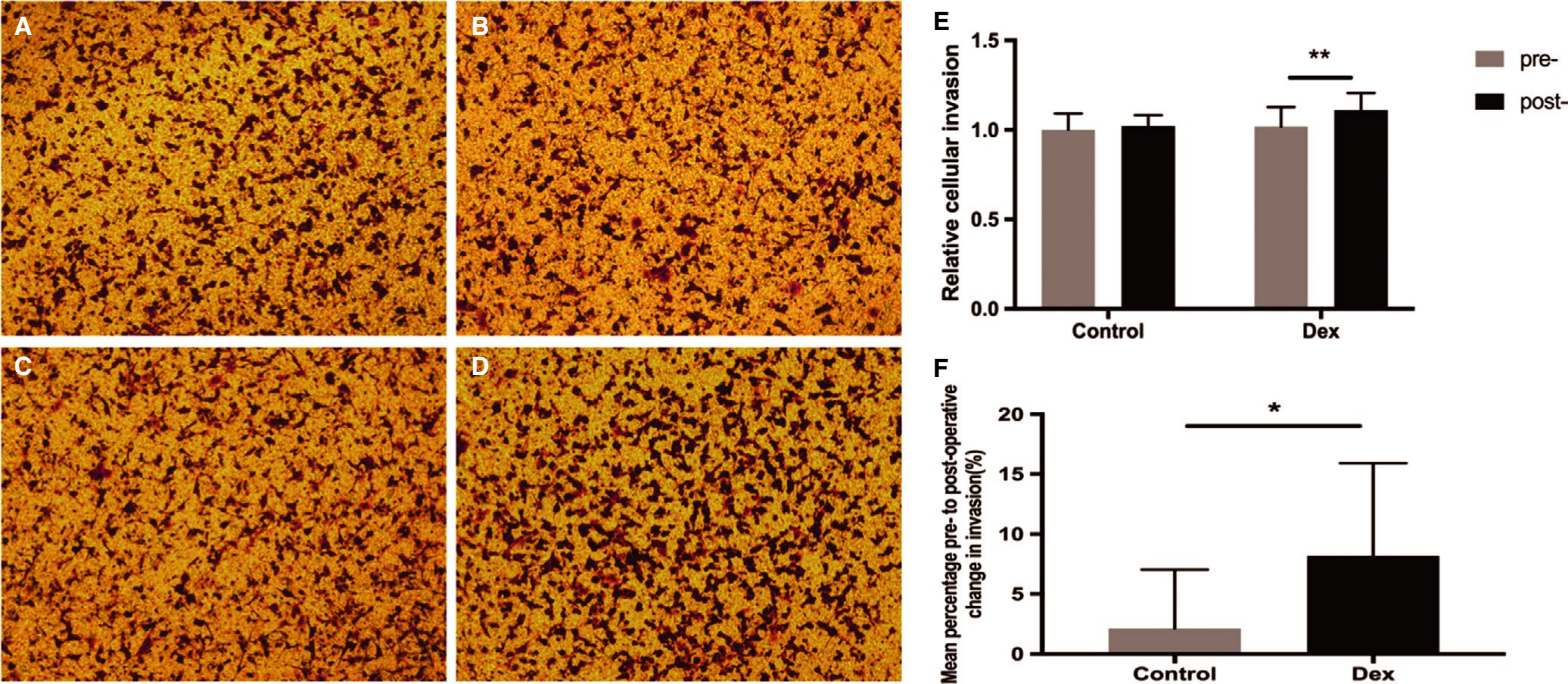
Serum from the dexmedetomidine group facilitated the cell invasion of MCF‐7. A, Serum from the control group, preoperatively; B, serum from the control group, 24 h postoperatively; C, serum from the Dex group, preoperatively; D, serum from the Dex group, 24 h postoperatively. Original magnification, 100×. E, Graphical representation of cells that invaded the lower surface of the four groups. The value obtained with preoperative serum of control patients was considered 1.0 and the ratios relative to this are shown. F, Graphical representation of the mean percentage change from post to preoperative values in invaded cell numbers in the Dex group vs the control group. Values were expressed as mean ± SD. n = 11 in the control group. n = 13 in the Dex group. *, *P* < .05; **, *P* < .01. Dex = dexmedetomidine

Next, we analyzed the migration and invasion activities of MCF‐7 cells, the other two malignant biological behaviors of cancer cells.^14^ In the migration assay, the number of cells migrating to the lower surface was significantly greater when postoperative Dex serum was added to the bottom chamber compared with preoperative serum from patients in the same group (0.99 ± 0.05 pre‐Dex vs 1.14 ± 0.10 post‐Dex, *P* < .001), whereas no significant difference was observed in postoperative control serum vs preoperative control serum (1.00 ± 0.07 precontrol vs 1.03 ± 0.07 postcontrol, *P* = .22) (Figure 3A‐E). When comparing the mean percentage change from post to preoperative values in cell migration, there was a significant difference between the Dex group and control group (15.90% ± 10.57% in the Dex group vs 3.25% ± 4.94% in the control group, *P* = .0015) (Figure 3F). The results obtained in the invasion assay were the same as the migration assay (1.00 ± 0.09 precontrol vs 1.02 ± 0.06 postcontrol, *P* = .36; 1.02 ± 0.11 pre‐Dex vs 1.11 ± 0.10 post‐Dex, *P* < .001; 8.17% ± 7.75% change in the Dex group vs 2.13% ± 4.89% change in the control group, *P* = .04) (Figure 4A‐F).

## 4 DISCUSSION

In this prospective, randomized, and controlled clinical trial recruiting patients undergoing surgery for primary BC, postoperative serum from those who received Dex administration during surgery was associated with significantly increased cell proliferation, migration, and invasion compared with preoperative serum when used to culture BC cell line MCF‐7 cells in vitro. Since these cell functions are tightly connected with the ability of tumors to progress into an invasive and metastatic phenotype, our results indicate that perioperative Dex might be used cautiously in BC patients.

Dex is a highly selective α2‐AR agonist, yielding an α2/α1 ratio of 1620.^14^ Since utilization of Dex in BC surgeries significantly increases the ratio of patients discharged on the same day, reduces the postoperative consumption of analgesia, and downregulates postoperative side effects, such as nausea, vomiting, and bleeding,^15^, ^16^ it is now widely used in BC surgical patients. However, much evidence has suggested that activating α2‐AR may induce BC relapse or metastasis.^4^, ^8^ Therefore, whether Dex affects BC progression has attracted great attention in recent years. Several studies have investigated the role of Dex in the malignancy of BC cells and results suggested that Dex might have a negative impact on BC prognosis.^10^, ^11^, ^12^, ^17^ For example, Bruzzone et al incubated the mouse mammary tumor cell line MC4‐L5 with Dex for 2 days and found that cell proliferation was significantly enhanced.^7^ Xia et al reported that Dex both increased the proliferation, migration, and invasion ability of MDA‐MB‐231 cells in vitro and elevated the volume and weight of xenotransplant tumors in vivo.^12^ Besides BC, the negative effects of Dex on cancer metastatic progression in other tumor models have also been suggested. Recently, Lavon and colleagues reported significantly increased tumor cell retention and metastasis number when subhypnotic and hypnotic/sedative dosages of Dex were administered in three clinically relevant models, including mammary adenocarcinoma in F344 rats, Lewis lung carcinoma in C57BL/6 mice, and colon adenocarcinoma in BALB/c mice.^18^

However, does Dex actually affect cancer prognosis in patients? Although priorities for clinical research in this area have been highlighted years ago,^19^ high‐quality clinical data have been lacking. Cata et al recently carried out a propensity score‐matched retrospective study on patients with non‐small‐cell lung cancer (NSCLC) and found that intraoperative use of Dex was associated with a significantly shorter overall survival (OS). They speculated that Dex could potentiate the progression of minimal residual disease during and immediately following surgery.^20^ Aside from this retrospective analysis, no randomized trial on the influence of Dex on clinical cancer patients has been reported.

The current study focused on the patients' serum as a marker of the overall effect of perioperative administration of Dex and investigated whether the serum from patients receiving Dex during primary BC surgery would make a difference in the BC cell malignancy. To allow a full action time of Dex, we collected the patients' serum at 24 hours postoperatively.^21^, ^22^ We chose the ER‐positive/HER2‐negative MCF‐7 cell line for this study because such tumors account for the largest subset, approximately 65%‐70%, of all BCs.^23^ Our finding that the mean percentage change from post to preoperative values in cell proliferation, migration, and invasion was significantly higher in the Dex group than in the control group has potential clinical implications. Sustaining proliferation is the most fundamental trait of cancer cells, as well as local invasion and distant metastasis, all of which are directly related to pathological grades of malignancy.^24^ The more proliferative the cancer cells, the faster the cancer grows; the ability of cancer cells to undergo migration and invasion allows them to detach from the primary lesion by causing loss of cell‐cell adhesion or breakdown of capillaries and enter into the systemic circulation.^25^ Once arrival to the suitable metastatic sites such as bone and lung, tumor cell extravasation and metastasis would occur. Therefore, our results indicate that perioperative Dex may worsen the prognosis of BC patients.

The mechanisms of Dex modulating the patients' serum milieu to a direction that favors the malignancy of MCF‐7 cells are unknown at present. Recently, several researchers have reported that Dex could negatively affect the function of both murine and human dendritic cells (DCs) by suppressing the maturation and migration of DCs.^26^, ^27^ DCs are the most potent antigen‐presenting cells and play a pivotal role in perioperative antitumor immunity by stimulating the clonal proliferation of cognitive lymphocytes, thus leading to the establishment of adaptive immunity.^26^, ^27^, ^28^ Thus, suppression of DC functions by Dex leads to immunosuppressive effects in patients' serum. Also other immune cells, such as macrophages, express α_2_‐AR on their surfaces.^29^, ^30^, ^31^ A recent experiment demonstrated that intravenous administration of Dex significantly decreased both the number and the activity of macrophages in the peripheral blood of mice.^32^ Whether Dex modulates the patients' serum milieu through alterations of immune reactions is currently under investigation in our lab.

Moreover, the molecular profile of the patients' serum may be altered by Dex. In mice treated with 10 μg kg^−1^ of Dex, serum levels of pro‐inflammatory cytokine TNF‐α were significantly increased.^17^ TNF‐α, which is supposed to induce hemorrhagic necrosis of tumors, is associated with enhanced tumor development and spread.^33^, ^34^, ^35^ It induces secretion of other pro‐inflammatory cytokines and chemokines, such as IL‐6, CXCL1, MCP‐1, and CCL2, which will subsequently stimulate tumor growth and home cancer cells to specific metastatic sites.^36^, ^37^, ^38^ Meanwhile, Szpunar and his collaborators demonstrated in an in vivo study that Dex promotes breast tumor progression through alterations in the extracellular matrix (ECM).^17^ Since matrix metalloproteinases (MMPs) are the main enzymes responsible for the degradation of the ECM,^39^ these findings indicate that Dex could possibly upregulate expression levels of serum MMPs. Notably, the main cells expressing MMPs in the blood, such as neutrophils and monocytes,^40^ both express α_2_‐AR.^41^, ^42^ Whether Dex modified the patients' serum through increasing the expression level of TNF‐α or other pro‐inflammatory cytokines or MMPs in serum warrants further investigation.

Our study has several limitations. First, the sample size is small. The approximate sample size of 22 was calculated based on an estimated difference of 15% between the two means, with a desired statistical power of 0.9 and a desired significance level of 0.05. Since the actual sample size of 24 and the observed difference of 22% in the primary outcome between the two groups are both a bit larger than the estimated value, the actual calculated power is 98%. Therefore, although with a small sample size, this study has high power and indicates that there is likely to be an effect of Dex on the prognosis of BC. Second, patients with or without NAC were included in the current study and treatment by NAC may be a confounder that will possibly interfere with the outcomes. However, we feel that the chances are small because (a) the ratio of patients receiving NAC was similar between the two groups (27% vs 38%, *P* = .68) and (b) both primary and secondary outcomes are the mean percentage change from post to preoperative values of the characteristic properties of MCF‐7 cells cultured in patients' serum in the current study. Therefore, even if NAC has some effects on patients' serum that will affect the malignancy of MCF‐7 cells, the effects will similarly exist in pre and postoperative serum, which will disappear during the calculation of “change.”

Recently, lowering the threshold for statistical significance from a *P* value of .05 to .005 has been proposed in medical research to reduce the misinterpretation of study results.^43^, ^44^ However, it has not been widely adopted because intense debates are ongoing regarding whether the *P* value should be lowered or not.^45^, ^46^ Nevertheless, we also evaluated the endpoints in the present study with a *P* value threshold of less than .005 to determine whether this new threshold could affect the conclusion. We found that the primary endpoint and one of the secondary endpoints, mean percentage change for migration from post to preoperative values, maintained the statistical significance, whereas the other secondary endpoint, the mean percentage change for invasion, had a *P* value less than .05 but greater than .005 and would be reclassified as “suggestive.” Therefore, the conclusion of the current study, ie, perioperative administration of Dex influences the serum milieu in a way that favors the malignancy of MCF‐7 cells, which can be retained even with a *P* value threshold of less than .005.

In summary, this prospective randomized clinical study shows for the first time that exposure of MCF‐7 cells to postoperative serum from patients receiving Dex during surgery, but not saline, had significantly higher proliferation, invasion, and migration than preoperative serum. The mean percentage change from post to preoperative values in all these cell malignant properties was also significantly larger in the Dex group compared with the control group. These findings suggest that the utilization of Dex during BC surgery may modulate the patients' serum milieu to a direction that favors the malignancy of MCF‐7 cells. However, since this study is a single‐center study with a relatively small sample size that provides indirect evidence, indicating the possibility of deleterious effects of Dex in the prognosis of BC patients, it should be viewed as a trial that identifies testable clinical problems. Large‐scale, multicenter, and prospective clinical trials investigating the long‐term oncological outcomes in BC surgery patients are needed to further clarify the effects of perioperative Dex on BC progression.

## CONFLICT OF INTEREST

The authors declare that they have no conflict of interest.

## AUTHOR CONTRIBUTIONS

Study conception and design: JT, JSL, DSS, and WFY. Study conduct: YL, JXS, TW, XYL, and YYD. Data analysis: JT, DSS, and HWD. Data interpretation: JT, JXS, JSL, and WFY. Manuscript preparation: YL, JT, DSS, and JSL

## Data Availability

The data that support the findings of this study are available on request from the corresponding author. The data are not publicly available due to privacy or ethical restrictions.
